# O-GlcNAc transferase influences the progression of degenerative cartilage disease in lumbar facet joint osteoarthritis through FoxO1 and EGR1

**DOI:** 10.1038/s41420-025-02732-1

**Published:** 2025-10-16

**Authors:** Chu Chen, Yongjing Gao, Guanhua Xu, Yuyu Sun, Guofeng Bao, Wei Liu, Dawei Xu, Kun Yuan, Zhiming Cui

**Affiliations:** 1 Department of Spine Surgery, The Second Affiliated Hospital of Nantong University, Nantong, Jiangsu China; 2 Spinal Degenerative Disease, Research Institute for Spine and Spinal Cord Disease of Nantong University, Nantong, Jiangsu China; 3 Department of Orthopaedics, The Second Affiliated Hospital of Nantong University, Nantong, Jiangsu China; 4 Institute of Pain Medicine, Institute of Nautical Medicine, Nantong University, Nantong, Jiangsu China; 5 Department of Orthopedics, Nantong Third People’s Hospital of Nantong University, Nantong, Jiangsu China; 6 Department of Orthopedics, Affiliated Cancer Hospital of Nantong University, Nantong, Jiangsu China

**Keywords:** Cytokinesis, Translational research

## Abstract

This study aimed to investigate the role of the O-GlcNAc transferase (OGT)/Forkhead Box O1 (FoxO1) signaling axis in age-induced facet joint osteoarthritis (FJOA) and its impact on chondrocyte homeostasis. Using a mouse model, the effects of OGT knockout on cartilage integrity in facet joints were examined. Molecular assays were conducted to analyze OGT’s influence on FoxO1 expression and its stability against ubiquitin-mediated degradation. In vitro studies with chondrocytes were performed to assess the impact of FoxO1 overexpression on extracellular matrix synthesis, while in vivo experiments were carried out to test the protective effects of FoxO1 overexpression on FJOA progression. Additionally, the functionality of ATDC5 cells and the regulation of EGR1 by the OGT/FoxO1 axis were evaluated. OGT knockout led to exacerbated cartilage degeneration in facet joints, promoting age-related FJOA. OGT was found to stabilize FoxO1 protein by amplifying its expression and preventing its degradation. The T627 site on FoxO1, along with other key sites (T317, S550, T648, S654), was identified as essential for FoxO1’s stability and transcriptional activity upon OGT overexpression. Elevated FoxO1 levels counteracted OGT deficiency in chondrocytes, enhancing extracellular matrix synthesis. In vivo, FoxO1 overexpression mitigated FJOA progression and chondrocyte apoptosis due to OGT deficiency. The OGT/FoxO1 signaling also regulated EGR1 expression, impacting ATDC5 cell function. Collectively, the OGT/FoxO1/EGR1 axis plays a critical role in maintaining chondrocyte homeostasis and protecting facet joint integrity, suggesting a potential therapeutic target for age-related FJOA and cartilage degeneration.

## Introduction

Facet joint osteoarthritis (FJOA) is a major degenerative disorder in the lumbar region, strongly associated with episodes of lower back pain (LBP) [1]. FJOA accounts for 15–45% of chronic LBP cases, and the prevalence of FJOA increases with age [2]. Imaging diagnostics often lack sensitivity in early diagnosis [3], resulting in patients presenting with significantly aggravated back pain only after severe damage to the facet joint cartilage has occurred [4]. Alterations in biomechanical architecture and imbalanced anabolic processes lead to the degradation of facet joints. This manifests as cartilage attrition, an increase in foundational bone (subchondral sclerosis), and can even cause FJOA-derived LBP [5].

The attachment of N-acetylglucosamine (UDP-GlcNAc) to proteins in the nuclear, mitochondrial, and cytoplasmic compartments, known as O-GlcNAcylation, is mediated by the coordinated actions of the enzymes O-GlcNAc transferase (OGT) and O-GlcNAcase. O-GlcNAcylation is highly responsive towards different stimuli, including trauma and cytokines, and has been found to be dysregulated in OA and other age-related degenerative diseases [6, 7]. However, O-GlcNAcylation plays a dual role in osteoarthritis (OA) by contributing to cartilage degeneration and synovial inflammation, while also potentially exerting anti-inflammatory effects through glucosamine (GlcN) regulation in synoviocytes [8, 9]. However, the potential regulatory mechanism of O-GlcNAcylation in arthritis needs to be further investigated.

Articular cartilage, crucial for joint cushioning, undergoes a shift in balance from disruptions in anabolic and catabolic signals, leading to FJOA [10]. Forkhead Box O (FoxO) proteins, evolutionarily conserved transcription factors, maintain chondrocyte homeostasis and stem cell populations during aging [11, 12]. Dysregulation of FoxO1 leads to disarray in downstream target genes, compromising cartilage integrity; these genes are implicated as contributing factors in various aspects of age-related OA [13]. Preliminary research by our group shows that mice with a cartilage-specific deletion of FoxO1 in their facet joints display pronounced FJOA characteristics, mediated by extracellular matrix synthesis and cell migration regulated by downstream target genes [14]. However, the mechanisms governing FoxO1 expression and transcriptional activity in FJOA remain unclear.

Changes in O-GlcNAcylation levels can impact numerous proteins, including FoxO1 and others with identified O-GlcNAcylation sites [15, 16]. The objectives of this study are threefold: first, to investigate whether reducing O-GlcNAcylation levels induces spontaneous FJOA; second, to examine OGT’s role in modulating FoxO1’s expression and transcriptional activity in chondrocytes; third, to delineate the target genes in the OGT/FoxO1 signaling pathway contributing to FJOA development. Hence, we examined the effect of the OGT/FoxO1 signaling axis on FJOA, assessed its functional roles in chondrocyte viability, migration, and apoptosis, and explored the molecular mechanisms underlying OGT/FoxO1-regulated cartilage degeneration, with the aim of identifying potential therapeutic targets for FJOA.

## Results

### OGT deletion induces spontaneous FJOA

**Fig. 1:**
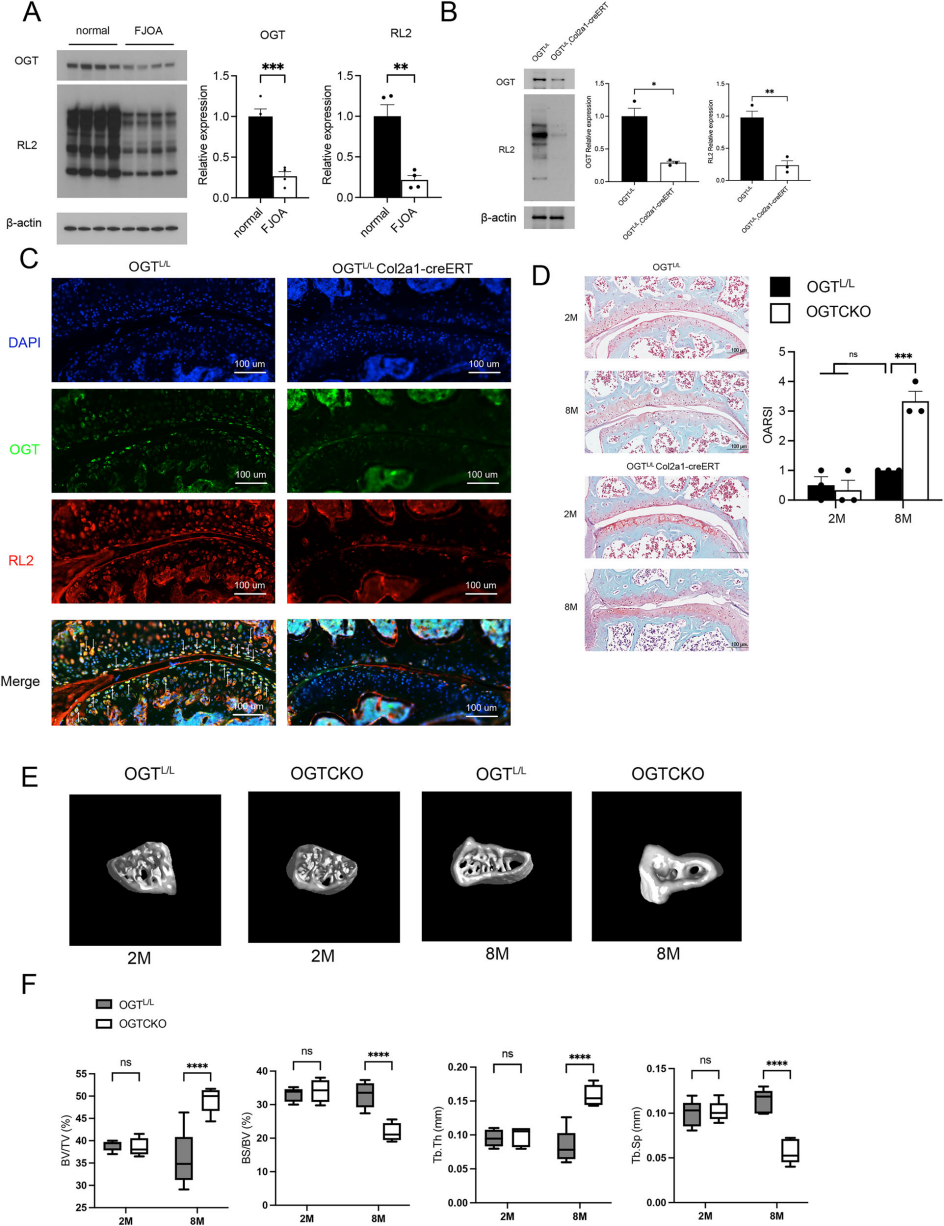
OGT deletion results in spontaneous FJOA. **A** Normal human facet joint cartilage and FJOA cartilage showing protein abundance of OGT and RL2 in western blot. **B** Comparison of protein abundance of OGT and RL2 in facet joint cartilage of OGTL/L and OGTL/L Col2a1-creERT mice, the full, uncropped Western blots are available as Supplementary Fig. S7. **C** Immunofluorescence staining of facet joint sections of OGT^L/L^ and OGT^L/L^ Col2a1-creERT mice. Blue indicates DAPI, green indicates OGT, and red indicates RL2. **D** Safranin O/fast green staining results of facet joint sections from OGT^L/L^ and OGT^L/L^ Col2a1-creERT mice. OARSI scores: Osteoarthritis Research Society International scores. **E** 3D micro-CT image of subchondral bone of the FJOA cartilage in the L5 superior articular process in OGTL/L and OGTL/L Col2a1-creERT mice. **F** Quantitative analyses of morphological parameters. Within these analyses, β-actin serves as a reference marker. Calibration lines represent 100 μm, Uncertainty markers: SEM. *P < 0.05. **P < 0.01. ***P < 0.001.

We collected human facet joint samples from cartilage exhibiting either negligible or only mild degeneration and from FJOA cartilage showing moderate to severe deterioration. Subsequently, we quantified the protein levels of OGT and RL2 (O-GlcNAc-specific antibody) in these samples. Protein levels for both OGT and RL2 were significantly reduced in the FJOA group compared to the normal group (Fig. 1A).

We then assessed the expression levels of OGT and RL2 proteins in the facet joint cartilage of OGT conditional knockout (OGTCKO) mice. Western blot analyses confirmed a significant decrease in the expression of OGT and RL2 in OGTCKO mice, indicative of effective OGT deletion and reduced O-GlcNAcylation levels (Fig. 1B). Additionally, immunofluorescence assays indicated a pronounced decrease in the counts of OGT and RL2-positive chondrocytes in the facet joint cartilage of OGTCKO mice (Fig. 1C).

By 8 months of age, OGTCKO mice displayed pronounced osteoarthritic changes in the L4/5 facet joint cartilage. We observed a significant decrease in safranin-O staining intensity, especially on the cartilage surface of the L5 superior articular process, accompanied with partial cartilage loss, and vertical clefts or erosion extending to the calcified zone. These findings contributed to heightened Osteoarthritis Research Society International (OARSI) scores (Fig. 1D). Conversely, such alterations were not observed in the OGT^L/L^ mice.

Subchondral sclerosis was widely recognized as a critical indicator marking the progression of OA [17]. We utilized micro-computed tomography (micro-CT) to delineate the micro-architectural modifications in the subchondral bone of OGTCKO mice spontaneously FJOA. Owing to the pronounced degenerative changes observed in the L5 superior articular process, we specifically selected this region for comprehensive analysis (Fig. 1E).

In 8-month-old OGTCKO mice, subchondral sclerosis was notably more advanced when compared to age-matched OGT^L/L^ mice. There was a significant increase in the ratio of bone volume to total tissue volume (BV/TV), accompanied by a marked decrease in the ratio of bone surface to volume (BS/BV). Furthermore, the trabecular width (Tb.Th) of the subchondral bone was substantially enlarged. In contrast, trabecular separation (Tb.Sp) demonstrated a decreasing trend, with the variations being statistically significant (Fig. 1F).

### OGT knockout influences movement behavior

In order to exclude other joint OA interference and focus exclusively on the impact of FJOA on animal movement behavior, we injected AAV2-cre into the facet joint articular cavity of the bilateral L4/5 level to deliberately knock out the OGT to induce FJOA, and detected catwalk-related indicators in mice at 8 months of age.

**Fig. 2:**
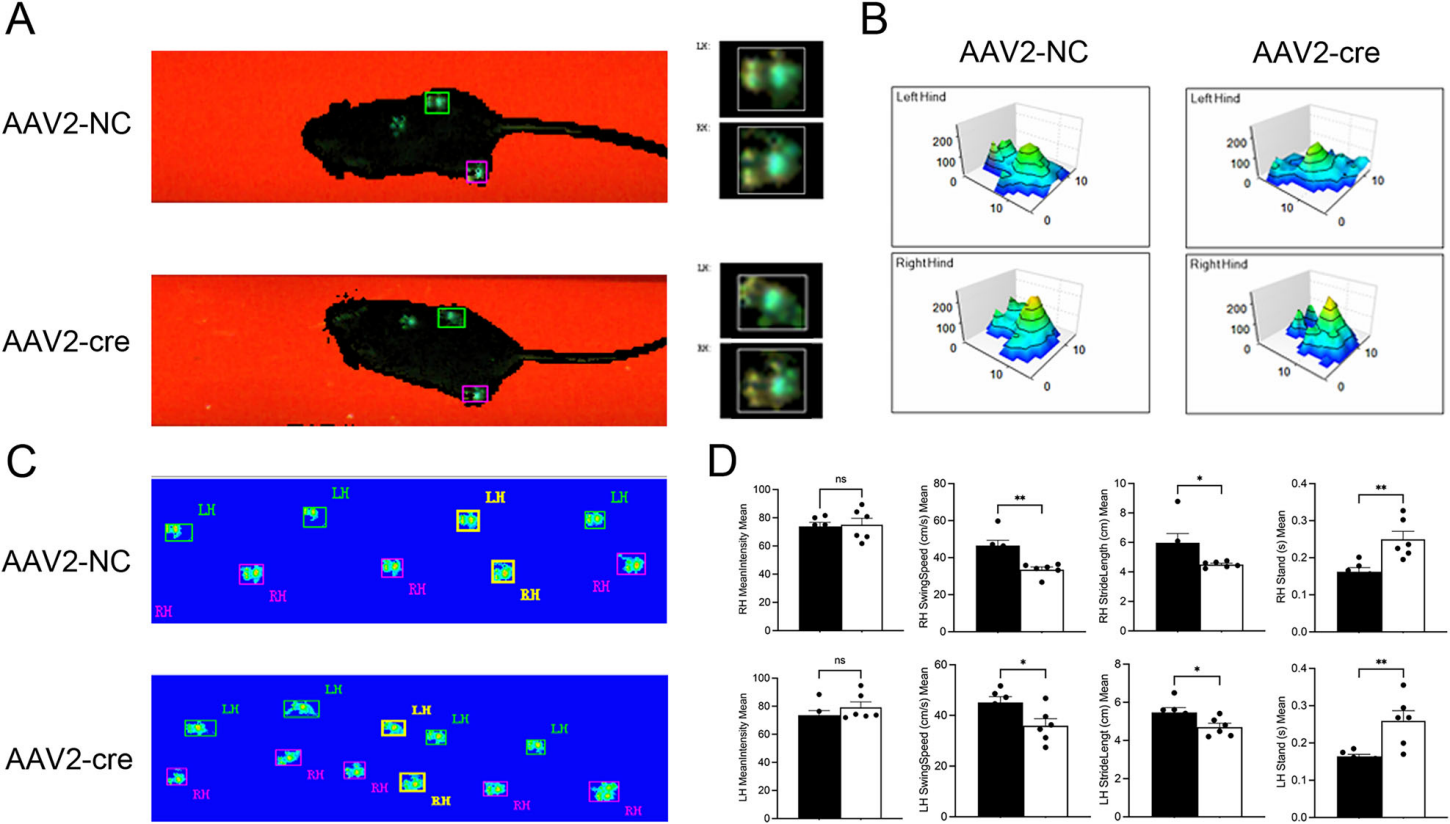
Catwalk gait analysis after OGT specific deletion in L4/5 bilateral facet joint cartilage. Representative **A**, **B** paw print images and **C** footprint intensities after injected with AAV2-NC or AAV2-cre in mice after knocking out the OGT. **D** CatWalk mean intensity, swing speed, stride length, and stand time in the left hind and right hind. Uncertainty markers: SEM. *N* = 6. *P < 0.05. **P < 0.01.

We utilized the Catwalk system for stride analysis to derive gait metrics from AAV2-NC and AAV2-cre mice aged 8 months (Fig. 2A–C). Catwalk gait analysis revealed a notable decrease in swing speed and stride length for the hind paws in 8-month-old AAV2-cre mice compared to the AAV2-NC group. Concurrently, an increased stand time was observed in the AAV2-cre group. Yet, the average strength of foot touch, measured as the average pixel intensity over the largest contact zone, exhibited no notable differences between the groups (Fig. 2D).

### OGT increases viability and migration ability of chondrocytes and inhibits chondrocytes apoptosis

**Fig. 3:**
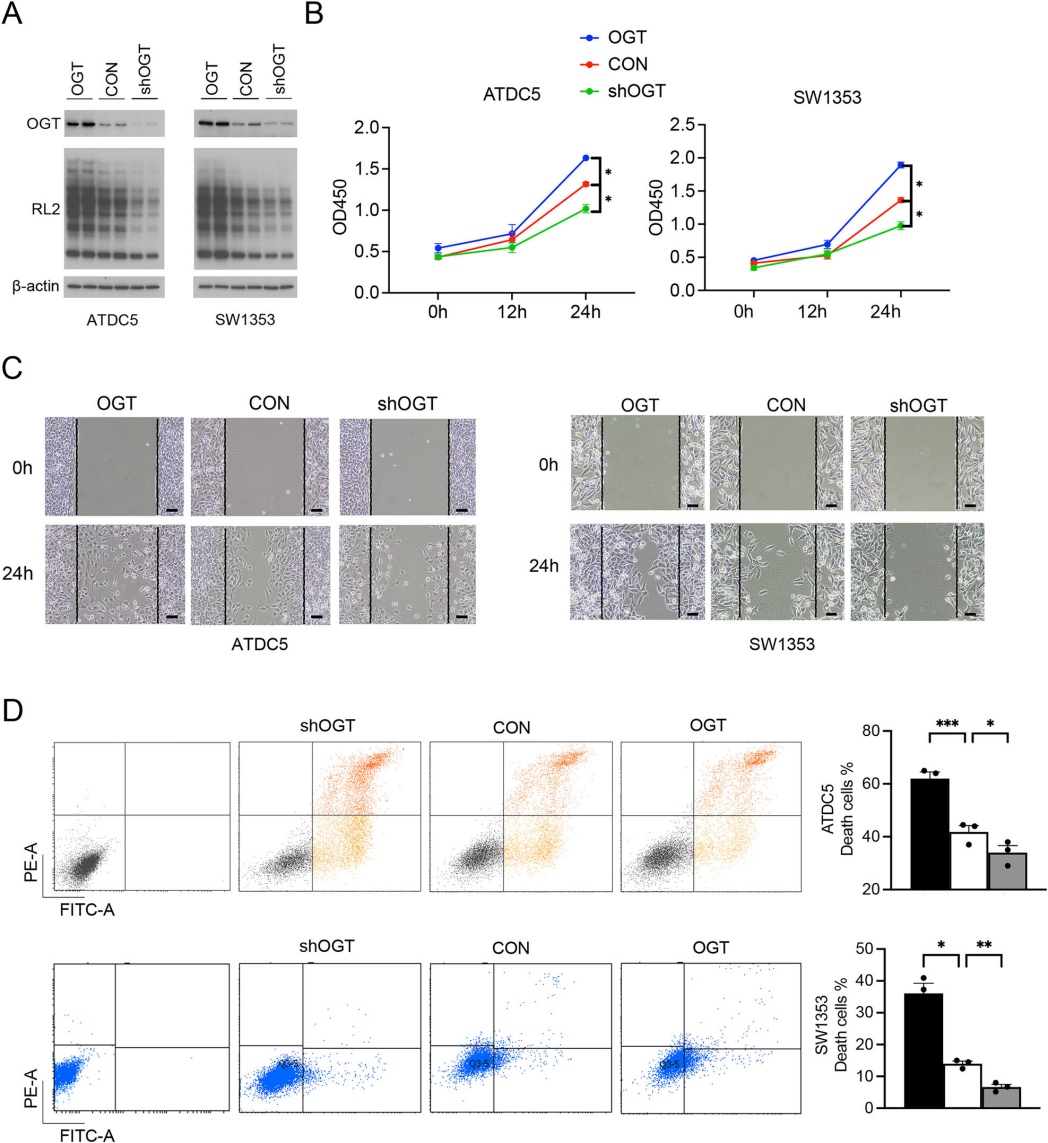
OGT knockdown or overexpression modulates chondrocytes viability, migration, and apoptosis. **A** Protein abundances of OGT and RL2 in ATDC5 and SW1353 cells transfected with shOGT or OGT-overexpressing lentivirus, The full, uncropped Western blots are available as Supplementary Fig. S8. **B** The viability of ATDC5 and SW1353 cells after transfection with shOGT or OGT-overexpressing lentivirus. **C** Migration ability of ATDC5 and SW1353 cells after transfection with shOGT or OGT-overexpressing lentivirus. **D** Apoptosis of ATDC5 and SW1353 cells after transfection with shOGT or OGT-overexpressing lentivirus. Within these analyses, β-actin serves as a reference marker. Calibration lines represent 100 μm, Uncertainty markers: SEM. *P < 0.05. **P < 0.01. ***P < 0.001.

Next, we examined the effects of OGT on chondrocyte behavior by transfecting chondrocytes ATDC5 and SW1353 with shOGT or OGT-overexpressing lentivirus. Reduced abundances of OGT and RL2 were observed in ATDC5 and SW1353 cells after OGT knockdown while increased abundances of OGT and RL2 were observed after OGT overexpression, indicating successful transfections with shOGT or OGT-overexpressing lentivirus (Fig. 3A). CCK-8 assay demonstrated that the viabilities of both ATDC5 and SW1353 cells were elevated after OGT overexpression but decreased after OGT knockdown (Fig. 3B). Wound scratch tests showed that in both ATDC5 and SW1353 cells, a smaller wound area was left at 24 h of cell culture after OGT overexpression while a larger blank space was observed after OGT knockdown, indicating that OGT increased chondrocyte migration (Fig. 3C). Results from Annexin V/PI flow cytometry revealed that there existed a smaller number of apoptotic cells in ATDC5 and SW1353 cells transfected with OGT-overexpressing lentivirus but a larger amount of apoptotic cells in ATDC5 and SW1353 cells transfected with shOGT (Fig. 3D).

### O-GlcNAcylation alters both the protein levels and transcriptional function of FoxO1

**Fig. 4:**
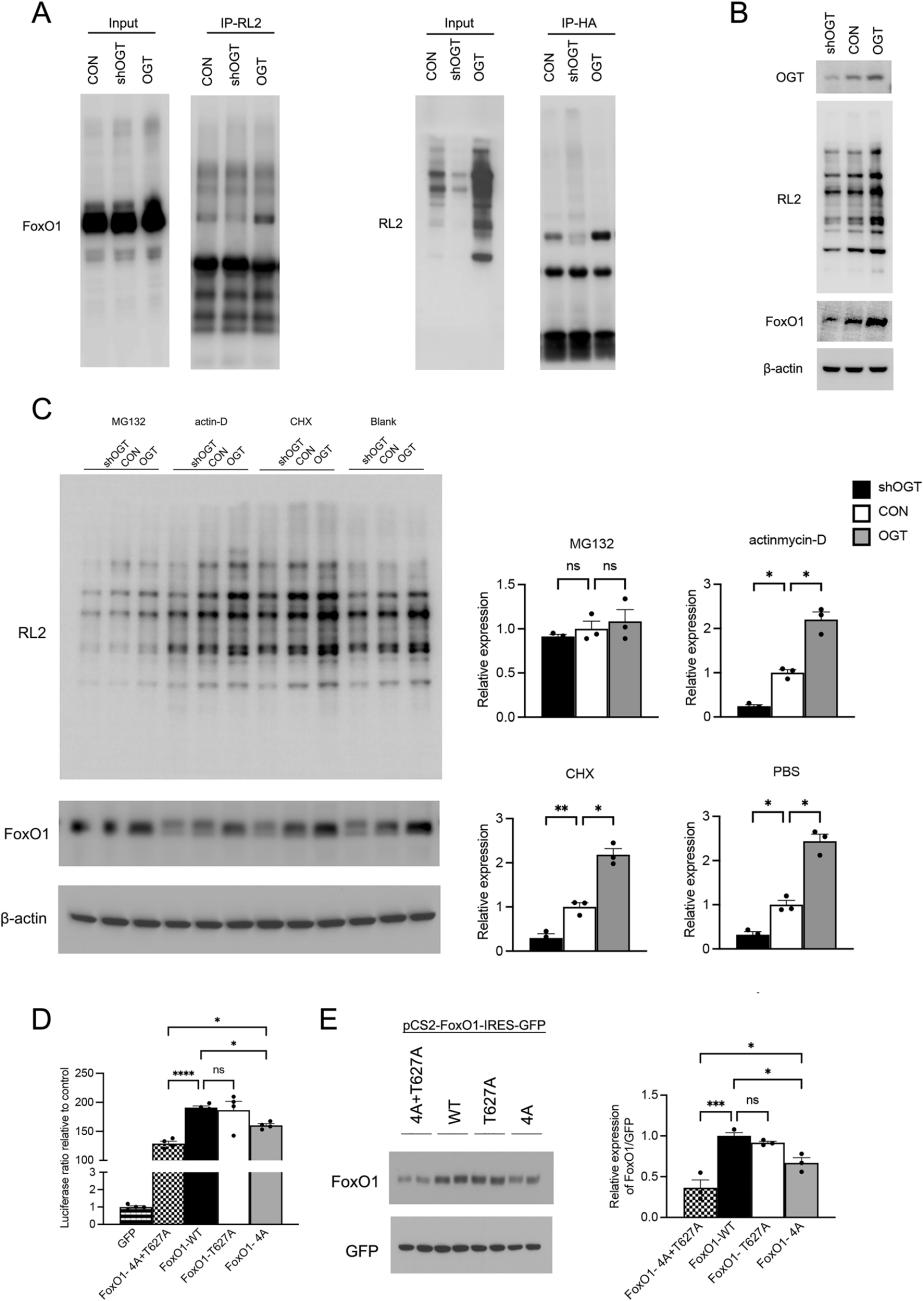
O-GlcNAcylation alters both the protein levels and transcriptional function of FoxO1. **A** Immunoprecipitation in HEK293 cells transfected with shOGT, control, and OGT-overexpressing lentivirus. **B** FoxO1 expression levels in ATDC5 cells transfected with shOGT, control, and OGT-overexpressing lentivirus. β-actin serves as a reference marker. **C** FoxO1 expression levels after treatment with MG132, actinomycin-D, CHX, and PBS (blank). **D** Changes in the transcriptional activation activity of FoxO1 on the IRE-luciferase (luc) reporter transfected with FoxO1-WT, FoxO1-T627A, FoxO1-4A, or FoxO1-4A + T627A during OGT overexpression in HEK293. **E** Changes of FoxO1 protein expression in cells transfected with FoxO1-4A + T627A, FoxO1-WT, FoxO1-4A, and FoxO1-T627A during OGT overexpression in HEK293, The full, uncropped Western blots are available as Supplementary Fig. S9. GFP is used as an internal control. Uncertainty markers: SEM. *P < 0.05. **P < 0.01. ***P < 0.001.

Immunoprecipitation assay revealed the interaction between OGT and transcriptional factor FoxO1 (Fig. 4A). Quantification of FoxO1 showed that FoxO1 increased with OGT overexpression and decreased upon OGT knockdown (Fig. 4B). Given that FoxO1 degradation occurs via the ubiquitin-proteasome pathway [18], we hypothesized that O-GlcNAcylation may inhibit its ubiquitination. To test this, we assessed FoxO1 protein expression using the proteasome inhibitor MG132, the transcript halting agent actinomycin-D, and the peptide synthesis deterrent cycloheximide. Results demonstrated that the ability of O-GlcNAcylation to modulate FoxO1 expression was blocked by MG132, suggesting O-GlcNAcylation might control FoxO1 expression by altering its ubiquitination (Fig. 4C).

To pinpoint the sites of O-GlcNAc modification, we conducted affinity purified mass spectrometry on HA-FoxO1 overexpressed in OGT-rich HEK293T cells. Following enrichment of HA-FoxO1 protein by HA-tag immunoprecipitation and in-gel protein digestion, mass spectrometry/electron transfer dissociation analysis culminated in pinpointing the probable O-GlcNAc modified site T627 (Supplementary Fig. 2).

In light of the ongoing debate in previous studies regarding the O-GlcNAcylation sites that influence FoxO1 protein expression and transcriptional activity, we generated the corresponding single (T627A), quadruple (Foxo1-4A), and combined 4A+T627A (FoxO1-5A) mutants in the highly conserved mouse Foxo1 cDNA sequence [19]. To validate that OGT regulates the ubiquitination level of FoxO1 through O-GlcNAc glycosylation modification, we co-transfected HA-FoxO1, HA-FoxO1-5A, and OGT, and immunoprecipitated with HA magnetic beads to verify the expression of FoxO1, RL2, and Ubiquitin proteins pulled down by the beads. The results showed that compared to the HA-FoxO1 group, the RL2 level was elevated and Ubiquitin was reduced in the HA-FoxO1 + OGT group. Conversely, when HA-FoxO1-5A + OGT was transfected, the RL2 level decreased, and Ubiquitin increased as compared to the HA-FoxO1 + OGT group. These findings indicate that OGT regulates the ubiquitination level of FoxO1 by O-GlcNAc glycosylation modification at the T317A, S550A, T648A, S654A, and T627A sites of FoxO1 (Supplementary Fig. 3). Upon expression in OGT-overexpressing HEK293T cells and subsequent WB analysis using RL2, we observed that while T627A mutation slightly reduced FoxO1 O-GlcNAc levels relative to the wild type, the 4A + T627A mutation further suppressed FoxO1 O-GlcNAcylation beyond the 4A alone (Supplementary Fig. 4). Our findings indicated the identification of a critical set of dominant O-GlcNAcylation sites. We subsequently evaluated their activation by OGT using a luciferase reporter assay. While the mutation of T627 to alanine did not significantly diminish transcriptional activation, the quadruple mutation (4A: T317A, S550A, T648A, and S654A) resulted in only a slight decrease in transcriptional activity, reducing it to approximately 80% (*P* < 0.05). However, combining the 4A mutations with the T627A mutation led to a pronounced drop in transcriptional activity, down around 50% (*P* < 0.001). This combined mutation also displayed a further reduction in transcriptional activity compared to the 4A mutation alone (*P* < 0.05) (Fig. 4D). In conclusion, our findings revealed that FoxO1 was O-GlcNAcylated at a combination of sites: T317, S550, T648, S654, and T627. To gauge the influence of these mutations on FoxO1 protein expression, we transfected HEK293 cells with four types of plasmids encoding GFP: 4A + T627A, WT, 4A, and T627A. Using GFP protein expression as an internal control, we evaluated FoxO1 protein expression levels, and the findings implied that, compared to the WT group, T627A modestly reduced FoxO1 protein expression, while 4A significantly decreased it. Moreover, compared to 4A, the 4A + T627A mutation further markedly suppressed FoxO1 protein expression (Fig. 4E).

### FoxO1 supplementation mitigates FJOA progression and chondrocyte changes

**Fig. 5:**
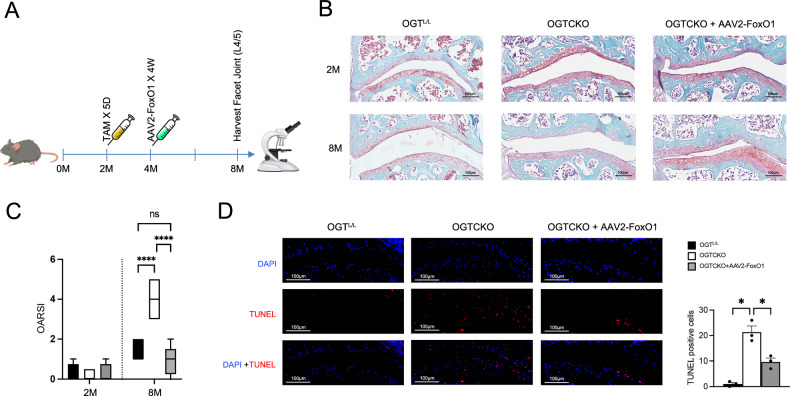
AAV2-FoxO1 mitigates FJOA progression and chondrocyte apoptosis in OGT knockout mice. **A** Schematic diagram of animal experiment. **B** Illustrative Safranin O/fast green tinting of sections taken from the facet joint cartilage of OGT^L/L^, OGTCKO, and AAV2-FoxO1 + OGTCKO mice. **C** OARSI scores of superior and inferior facets. IAP represents the inferior articular process, while SAP denotes the superior articular process. Calibration lines represent 100 μm. **D** Representative TUNEL staining images of OGT^L/L^ (Ctrl), OGTCKO, and AAV2-FoxO1 + OGTCKO mice at 8 months following TAM-induced OGT ablation and AAV2-FoxO1 enhancement of FoxO1 expression. Blue indicates DAPI and red indicates TUNEL. Calibration lines represent 50 μm. Uncertainty markers: SEM. *P < 0.05. **P < 0.01. ***P < 0.001.

To explore whether exogenous FoxO1 supplementation could counteract FJOA precipitated by OGT deficiency, the OGTCKO mice were administered AAV2-FoxO1 into the L4/5 facet joint up to the age of 8 months (Fig. 5A). Notably, FoxO1 supplementation appeared to lessen the occurrence of superficial cartilage damage, proteoglycan depletion, and cartilage wear (Fig. 5B). OARSI scores of both superior and inferior facets were significantly increased in the OGTCKO mice and reduced after exogenous FoxO1 supplementation (Fig. 5C). TUNEL staining further demonstrated that FoxO1 supplementation decreased the amount of apoptotic cells in OGTCKO mice (Fig. 5D).

**Fig. 6:**
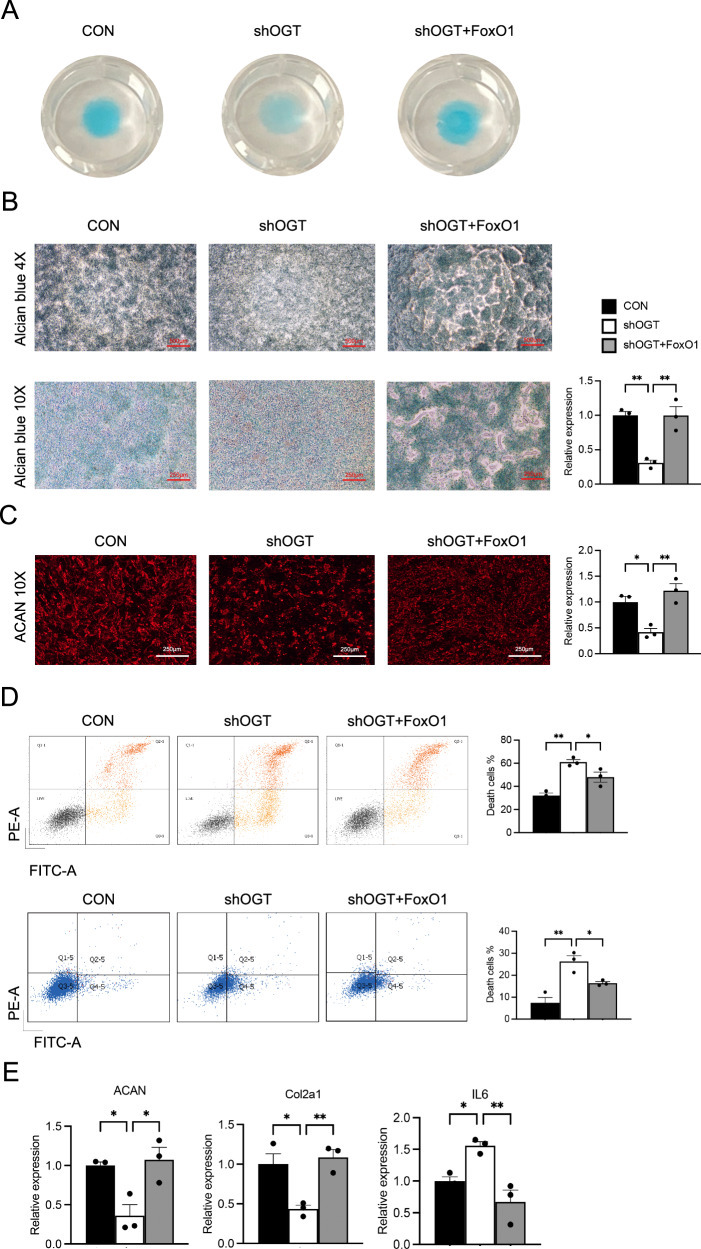
FoxO1 overexpression overcomes the effects of OGT knockdown on chondrocytes. **A** Representative micromass culture images of ATDC5 cells transfected with control, shOGT, and shOGT plus FoxO1-overexpressing lentivirus. **B** Alcian blue staining of ATDC5 cells transfected with control, shOGT, and shOGT plus FoxO1-overexpressing lentivirus. Scale bars indicate 500 μm and 250 μm. Quantification of the absorbance at 620 nm are summarized. **C** Immunofluorescence analysis of ACAN expression. Scale bars indicate 250 μm. **D** Apoptosis of ATDC5 cells after transfection with control, shOGT, and shOGT plus FoxO1-overexpressing lentivirus. **E** Gene abundances of ACAN, COL2A1, and IL6 in ATDC5 cells transfected with control, shOGT, and shOGT plus FoxO1-overexpressing lentivirus. GAPDH is used as an internal control. Uncertainty markers: SEM. *P < 0.05. **P < 0.01. ****P < 0.0001.

Micromass culture and Alcian blue staining showed that elevated FoxO1 could recover reduced proteoglycan synthesis induced by reduced OGT (Fig. 6A, B). The relative expression of ACAN-positive chondrocytes in shOGT-transfected ATDC5 cells was increased after co-transfection with FoxO1-overexpressing lentivirus (Fig. 6C). After FoxO1 overexpression, the amount of shOGT-induced cellular apoptosis was reduced as well (Fig. 6D). RT-PCR quantification showed that OGT knockdown reduced the expression of ACAN and COL2A1 in ATDC5 cells, while FoxO1 overexpression rescued ACAN and COL2A1 gene abundances. On the contrast, IL6 gene expression in ATDC5 cells was increased after OGT knockdown but decreased after FoxO1 overexpression (Fig. 6E).

### OGT/FoxO1 affects chondrocytes by regulating EGR1

**Fig. 7:**
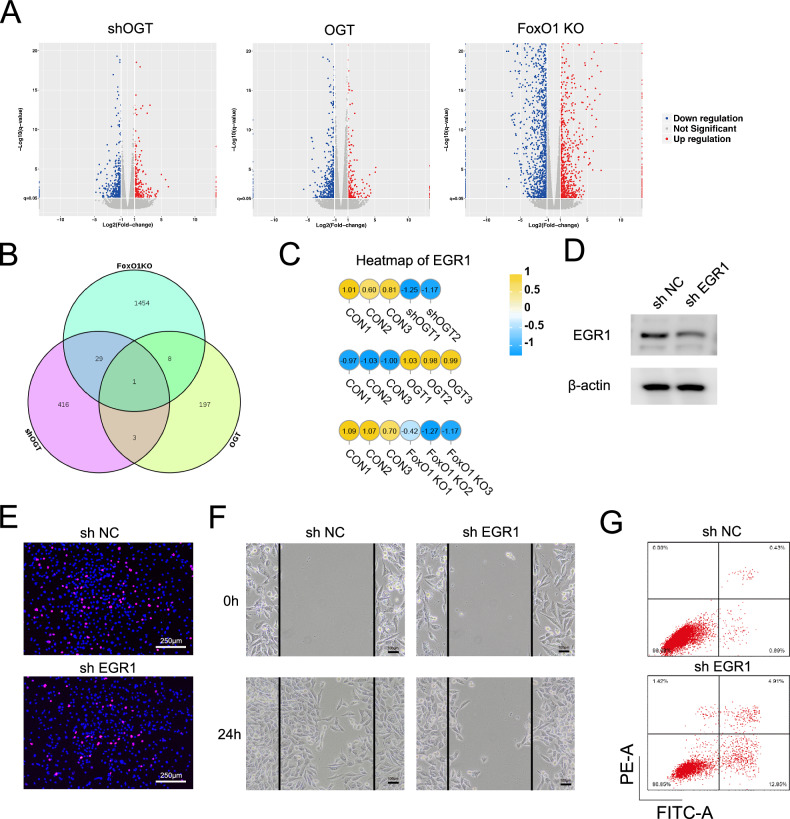
Promotion of chondrocyte proliferation, migration, and inhibition of apoptosis by OGT/FoxO1 downstream target gene EGR1. **A** Dispersion diagrams showcasing variances in gene expression within SW1353 cells post shOGT and OGT-overexpressing lentivirus transfection as well as in facet joint cartilage of FoxO1 knockout mice. Red hues highlight notably elevated genes, while blue tones mark those that are substantially reduced. **B** Venn diagram of downregulated genes in SW1353 cells transfected with shOGT and FoxO1 knockout mice as well as upregulated genes in SW1353 cells transfected with OGT-overexpressing lentivirus. **C** Heatmap showcasing EGR1 RNA-seq expression variations. Amber shades signify increased levels, while azure shades denote decreased ones. **D** Protein abundance of EGR1 in ATDC5 cells transfected with shEGR1. β-actin is used as an internal control, the full, uncropped Western blots are available as Supplementary Fig. S10. **E** Proliferation of ATDC5 cells after transfected with shEGR1. Red color indicates EdU-positive cells and blue color indicates nucleus. Scale bars indicate 250 μm. **F** Migration ability of ATDC5 cells after transfected with shEGR1. Scale bars indicate 100 μm. **G** Apoptosis of ATDC5 cells after transfected with shEGR1.

To elucidate the molecular mechanism induced by OGT/FoxO1, we collected RNA samples from shOGT and OGT-overexpressing SW1353 cells, as well as FoxO1 knockout facet joint cartilage and conducted RNA sequencing. In the shOGT group, we identified 225 upregulated and 449 downregulated genes. In the OGT-overexpressing group, 209 genes had elevated expression, while 478 displayed reduced levels. In the FoxO1 knockout group, 922 genes exhibited increased expression, while 1495 showed a decline (Fig. 7A). A joint analysis of differentially expressed genes (DEGs) in the shOGT, OGT-overexpressing, and FoxO1 knockout groups discovered a common differentially expressed gene among three groups, i.e., EGR1 (early growth response 1) (Fig. 7B). EGR1 was found to be downregulated in both the shOGT and FoxO1 knockout groups but upregulated in the OGT-overexpressing group (Fig. 7C and Supplementary Fig. 5A). Also, RT-PCR quantification showed that OGT knockdown reduced the expression of EGR1 in ATDC5 cells while FoxO1 overexpression rescued EGR1 gene abundances. We evaluated EGR1 expression levels and the findings implied that, compared to the CON group, overexpression FoxO1 significantly increased EGR1 expression levels, however 4 A modestly reduced EGR1 expression levels, while compared to 4A, the 4A + T627A mutation further markedly suppressed EGR1 expression levels (Supplementary Fig. 5B, C).

Functional roles of EGR1 on chondrocytes were examined by transfecting with shEGR1 to reduce EGR1 abundance (Fig. 7D). EdU proliferation assay, wound healing assay, and TUNEL staining showed that the expansion and movement of chondrocytes were suppressed while the apoptosis was elevated after shEGR1 transfection (Fig. 7E–G).

## Discussion

LBP is frequently categorized as nonspecific, yet it’s often linked to specific conditions like intervertebral disc degeneration (IDD) and FJOA [20]. Although the intervertebral disc and facet joints are closely biomechanically related, there has been a historical focus on differentiating IDD from facet joint OA. While these conditions often occur together and IDD is typically seen before facet joint OA [21, 22], FJOA can occur in isolation from disc degeneration, affecting about 20% of those with the condition [3, 23]. This suggests that localized mediators of spinal degeneration can vary. In this subset of patients, lumbar facet joint-mediated pain (facetogenic LBP) is the primary cause [1, 24]. Due to the low sensitivity of imaging diagnostics and the absence of specific disease biomarkers [25], patients often do not seek treatment until significant facet joint cartilage destruction has occurred.

The causes and underlying mechanisms of spontaneous FJOA are still not fully understood till now.

Our current study is the first to establish a connection between O-GlcNAcylation, particularly OGT, and the pathogenesis of FJOA as well as cartilage homeostasis. Our results indicated that as FJOA progressed, cartilage degeneration led to reduced levels of O-GlcNAcylation and OGT in chondrocytes. This observation appears to contradict the elevated O-GlcNAcylation levels found in articular cartilage of knee OA [8]. However, the transcriptomic profiles of degenerated cartilage in OA may vary significantly across anatomical locations due to unique gene expression patterns shaped by the specific biomechanical demands and structural functions of each joint. For example, synovial fibroblasts in the shoulder, hand, and knee exhibit distinct RNA expression profiles based on their location, rather than disease status alone [26]. Our previous sequencing of human FJOA-degenerated cartilage demonstrated a decrease in OGT gene expression [27]. Consistent with this trend, protein levels of both OGT and RL2 were significantly lower in the FJOA group compared to the normal group in this study.

Due to the lethality of OGT gene deletion during ontogeny [28], we utilized Col2a1-creERT knock-in mice [29] to specifically investigate the role of OGT in maintaining mature articular cartilage and its involvement in FJOA pathogenesis. In the OGTCKO-induced FJOA model, we observed significant changes in bone morphometrics, aligning with previous studies showing that conditional gene knockout in cartilage of mice induces osteoarthritic subchondral bone remodeling [11]. Abnormal subchondral bone remodeling, angiogenesis, and sensory nerve innervation directly or indirectly contribute to cartilage destruction and pain in OA [30, 31]. Studies have shown that increased blood vessel formation and nerve innervation are also present in degenerated lumbar facet joints, particularly in the subchondral bone region [32]. Previous studies have primarily utilized indirect methods such as the withdrawal test [33], grip force test, vocalization threshold in algometer test, and straight leg raising test [34] to assess pain augmentation in FJOA-related LBP. A meta-analysis indicated that there is moderate-to-strong evidence showing that individuals with persistent LBP demonstrate altered gait patterns compared to back-healthy controls. In this study, we specifically induced FJOA by injecting AAV2-cre into the lumbar facet joints, intentionally limiting the impact to these joints without affecting others. To assess functional outcomes, CatWalk analysis was performed. The results demonstrated a significant reduction in swing speed and stride length of the hind paws in the AAV2-cre group, along with an increase in stand time. Interestingly, the mean intensity of paw placement remained comparable between the AAV2-cre group and controls, suggesting that while motor function was altered, overall paw pressure remained unaffected. This suggests that the motor impairments are not solely due to a lack of plantar force but are more likely related to alterations in neural control or coordination abilities.

O-GlcNAcylation homeostasis occurs within an “optimal zone” of signaling where O-GlcNAcylation levels are well-buffered [35]. FoxO1, recognized as a key factor in promoting longevity, plays a critical role in preserving chondrocyte homeostasis. Its regulation is highly dependent on various post-translational modifications (PTMs), which modulate FoxO1’s stability, activity, and localization within the cell [36]. The OGT/FoxO1 signaling axis is pivotal in regulating chondrocyte function and mitigating FJOA. OGT overexpression enhances chondrocyte viability, migration, and reduces apoptosis, while OGT knockdown results in decreased viability and increased apoptosis. In in vivo studies, FoxO1 supplementation in OGT-deficient (OGTCKO) mice alleviated FJOA by reducing cartilage damage, proteoglycan loss, and apoptosis, as indicated by improved OARSI scores and TUNEL staining. In vitro, FoxO1 restored proteoglycan synthesis and upregulated key cartilage-related genes (ACAN and COL2A1), while downregulating the pro-inflammatory IL6. This axis plays a critical role in maintaining cartilage health by modulating extracellular matrix synthesis and inflammatory responses, making it a promising target for FJOA therapy.

Recent findings indicate a novel interaction between O-GlcNAcylation and the regulation of FoxO1 protein expression [37]. However, the precise mechanisms governing this relationship remain unclear [19]. In this study, we explored how O-GlcNAcylation, regulated by OGT, modulated FoxO1. Using qRT-PCR quantification, we found that the mRNA abundance of FoxO1 was not significantly altered (unpublicated data). Therefore, we proposed that O-GlcNAcylation might inhibit FoxO1 ubiquitination, a theory supported by our results using the proteasome inhibitor MG132. Moreover, the identification of a new O-GlcNAc modification site, T627, and the combination of T627A and T317A, S550A, T648A, and S654A mutations (4A) suppressed FoxO1 O-GlcNAcylation beyond the 4A mutation alone. Notably, the newly identified T627 site in FoxO1’s transactivation domain [38], in conjunction with traditional sites such as T317A, S550A, T648A, and S654A, plays a synergistic role crucial for target gene transcription.

At last, the study used RNA sequencing to examine OGT/FoxO1’s impact on genes, emphasizing EGR1’s role. Reduced EGR1 hindered chondrocyte growth and increased apoptosis, indicating its key role in chondrocyte function and its relationship with OGT/FoxO1. These findings provide new perspectives for understanding the molecular mechanisms of FJOA, revealing EGR1 as a crucial effector molecule downstream of the OGT/FoxO1 signaling pathway that regulates cartilage homeostasis imbalance. Its aberrant expression may represent a key factor in facet joint cartilage degeneration. Future studies will focus on elucidating the precise regulatory network of EGR1 in FJOA, including upstream kinase/phosphatase cascades and epigenetic modification mechanisms, which will establish the theoretical foundation for developing EGR1-targeted precision therapies.

In summary, this study unveiled the involvement of OGT and O-GlcNAcylation in the onset of FJOA and cartilage homeostasis. Meanwhile, it identified new therapeutic targets and elucidated the importance of PTMs in disease pathology. Future research should aim to address the remaining questions on the detailed mechanisms and the role of specific O-GlcNAcylation sites on FoxO1.

**Fig. 8:**
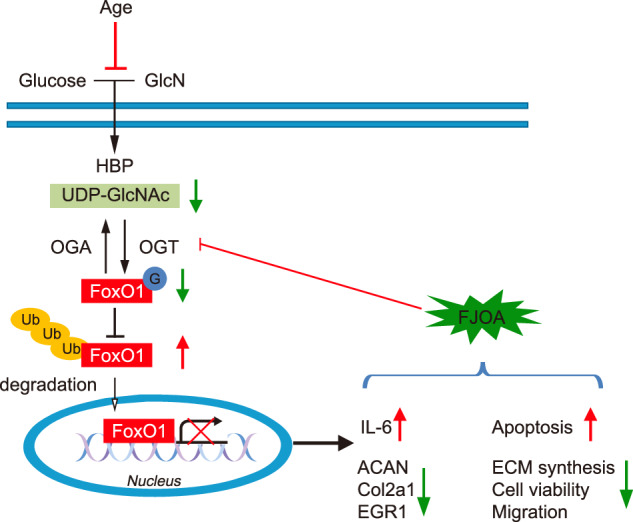
Schematic diagram of the OGT/FoxO1 signaling pathway mechanism.

Decreased OGT is associated with diminished protein expression and transcriptional activity of FoxO1. OGT/FoxO1 further regulates its downstream targets, including Col2a1, ACAN, EGR1, and IL-6, thereby mediating chondrocyte behavior (Fig. 8). Further research is needed to better understand this mechanism for clinical applications.

## Materials and methods

This protocol was approved by the Human Ethics Committee of the Second People’s Hospital of Nantong University (approval no. 2022KT065), and informed consent was obtained from the patients. A total of 8 lumbar facet joint tissues were collected from donors (4 cases in the control group and 4 cases in the FJOA group). The control group consisted of L4-L5 facet joint tissues from patients undergoing internal fixation surgery for vertebral fractures (MRI-based cartilage degeneration grading: Grade 0, *n* = 2; Grade 1, *n* = 2); the FJOA group consisted of L4-L5 facet joint tissues from patients undergoing surgery for lumbar spinal stenosis caused by FJOA (MRI-based cartilage degeneration grading: Grade 2, *n* = 3; Grade 3, *n* = 1).

The animal experiments involved in this study were approved by the Jiangsu Administration Committee for Experimental Animals (approval no. S20220221-006). Col2a1-creERT transgenic mice (C57BL6/J background) were purchased from GemPharmatech Co., Ltd. (Strain ID: T014706), and OGTL/L mice were purchased from The Jackson Laboratory (Strain number: 004860). The specific number of mice used in each experimental group (including age, sex, and genotype distribution) is detailed in Table 1.

**Table 1:** Information of mouse samples.

Fig	Postnatal age	Control	OGTL/LCol2a1-creERT
Fig. 1B	8-month-old(6 months after tamoxifen)	3 (all male)	3 (all male)
Fig. 1D	2-month-old	3 (all male)	3 (all male)
	8-month-old(6 months after tamoxifen)	3 (all male)	3 (all male)
Fig. 1E,F	2-month-old	3 (all male)	3 (all male)
	8-month-old(6 months after tamoxifen)	3 (all male)	3 (all male)
Fig. 2	8-month-old(Starting at 2 months of age, inject AAV2-Cre once a week for four consecutive weeks)	6 (all male)	6 (all male)
Fig. 5	2-month-old	3 (all male)	3 (all male)
	8-month-old(Starting at 2 months of age, administer TAMvia intraperitoneal injection for five consecutive days. At 4 months of age, begin intra-articular injection of AAV2-Cre once a week for four consecutive weeks)	3 (all male)	3 (all male)

### Safranin O and fast green staining

The spinal facet joints of humans and murine were fixed with 4% paraformaldehyde (p0099, Beyotime, Shanghai, China), followed by decalcification using JYBL-I decalcifying solution (G2470, Solarbio, Beijing, China) until the tissue became soft enough to be penetrated by a syringe. After dehydration, clearing, infiltration with paraffin, and embedding, paraffin sections were prepared. The sections were stained with Safranin O and Fast Green to distinguish cartilage from bone, and the staining intensity was quantitatively analyzed using ImageJ Fiji software. The OARSI scoring framework was employed to assess osteoarthritic changes.

### Western blot

Total proteins from human and murine spinal facet joints cartilage and cells were extracted using RIPA lysis buffer (p0013B, Beyotime, Shanghai, China) and subsequently denatured by adding Laemmli buffer and heating. The proteins were separated by SDS-PAGE on a 10% gel. After transfer to membranes and blocking, the membranes were incubated overnight at 4 °C with primary antibodies, including anti-FoxO1 (rabbit, 18592-1-AP, Proteintech), anti-OGT (rabbit, 11576-2-AP, Proteintech), anti-RL2 (mouse, MA1-072, Invitrogen), anti-EGR1 (rabbit, 22008-1-AP), and anti-β-actin (rabbit, 20536-1-AP, Proteintech). Wash the membrane 3 times with TBST, then incubate the membrane with secondary antibody at room temperature in the dark for 1 h. Finally, develop with ECL (34580, Thermo, Massachusetts, USA), visualize the image using a gel imaging system (FL1500, Thermo Fisher Scientific, MA, USA), and analyze band intensity with ImageJ.

### Generation of OGT knockout mice

Mice with the Col2a1-creERT transgene on a C57BL6/J background were obtained from GemPharmatech Co., Ltd (Strain ID T014706), while OGT^L/L^ mice were acquired from The Jackson Laboratory (Strain number: 004860). All mice were kept until two months of age and administrated with Tamoxifen (sourced from Sigma-Aldrich), applying a regimen of 100 mg/kg body weight across a successive 5-day period through intraperitoneal injection. Genetic identification was performed through PCR on DNA samples from the mice’s tails. Littermates homozygous for the floxed OGT gene without Cre recombinase expression were used as controls for the Col2a1 creERT-OGT knockout mice.

For genotyping PCR, the employed primers were delineated below: Col2a1-creERT: forward (F),5′-GGGCAGTCTGGTACTTCCAAGCT-3′, reverse (R), 5′-GCTAGGACAGGGTCTGGTGAATT-3′; WT: F, 5′-GGCCTCCAAGTCTTGACAGTAGATT-3′, R, 5′-TTGTGGGTCTTCCACCTTTCTTC-3′; OGT^L/L^: F, 5′-GACCACCTTTTTAGCCTCCTG-3′, R, 5′-AGGGAAGGGACTTAGGAATGA-3′.

The creERT system is sensitive to endogenous estrogen [39], so we chose male mice as the experimental subjects.

For the creation of L4/5 bilateral facet joint cartilage-specific OGT knockout mice, we utilized OGT^L/L^ male mice aged 2 months for the AAV2-cre intra-articular injection procedure. Specifically, recombinant AAV2 carrying FoxO1 (AAV2-cmv-m-FoxO1) was procured from (HanBio, China) at a titer of 1 × 10^12^ vg/mL. A 5 µL volume of the viral construct was delivered into the L4/5 lumbar facet joints of OGT knockout (KO) mice using a microinjector, administered once weekly for four consecutive weeks. Tissues were harvested at 8 months of age. OGT^L/L^ male subjects [18–22 g; *n* = 6 rats/group (*n* = 12 FJs/group)] were anesthetized with 2.0% isoflurane carried in oxygen, arranged in a prone stance, in alignment with past references.

### Quantitative reverse transcription polymerase chain reaction (qRT-PCR)

Total RNA was extracted using the RNeasy Plus Kit (Qiagen), and then cDNA was synthesized using SuperScript™ III SuperMix (11752250, Invitrogen) with RNA as the template. Subsequently, specific primers for the target RNA (primer sequences in Table 2) were selected and PCR amplified by pre-designed fluorescent labeling probes. During real-time PCR monitoring, an increase in fluorescence signal was proportional to the accumulation of cDNA, thereby achieving quantification of RNA expression. Relative expression was calculated using corrected internal reference genes and the 2^^^^−ΔΔCt^ method, each experimental step involving at least three biological repetitions.

**Table 2:** Primer sequences.

Primers	Sequences
ACAN-F	3′-CAGGCTATGAGCAGTGTGATGC-5′
ACAN-R	3′-GCTGCTGTCTTTGTCACCCACA-5′
COL2A1-F	3′-GCTGGTGAAGAAGGCAAACGAG-5′
COL2A1-R	3′-CCATCTTGACCTGGGAATCCAC-5′
IL-6-F	3′- TACCACTTCACAAGTCGGAGGC -5′
IL-6-R	3′- CTGCAAGTGCATCATCGTTGTTC -5′
GAPDH-F	3′-CATCACTGCCACCCAGAAGACTG-5′
GAPDH-R	3′-ATGCCAGTGAGCTTCCCGTTCAG-5′

### Immunofluorescence

Add 0.3% Triton X-100 permeabilization solution to the sections and incubate at room temperature for 5 min. Block with goat serum at room temperature for 30 min. After discarding the blocking solution without washing, add primary antibodies (OGT and RL2) and incubate overnight at 4 °C. Wash the samples three times with PBS, then add Alexa Fluor 488 (A11008; Thermo Fisher, MA, USA) and incubate in the dark for 1 h. Add DAPI(R37606, Thermo Fisher, MA, USA) for nuclear staining and incubate in the dark for 5 min. Finally, mount with antifade reagent and observe under a microscope (Mshot MF53, Mshot-Mingmei, Guangzhou, China).

### ATDC5 and SW1353 cell culture and treatment

ATDC5 mouse Chondrogenic Cell Line, sourced from RIKEN, the Japanese Institute of Physical and Chemical Research, catalog number RCB0565. SW1353 human Chondrosarcoma Cell Line, obtained from American Type Culture Collection, catalog number HTB-94. Revive ATDC5 and SW1353 cells and culture them in DMEM (C11995500BT, GIBCO, USA) supplemented with 10% fetal bovine serum (FBS) (AC03L055, Life-iLab, Shanghai, China) and 1% penicillin-streptomycin (C0222, Beyotime, Shanghai, China), under 5% carbon dioxide at a constant temperature of 37 °C. Add 1% Insulin-Transferrin-Selenium (41400045, Thermo Fisher Scientific, MA, USA) when the cell culture approaches sub-confluence to stimulate chondrogenic differentiation. Construct lentiviral vectors containing short hairpin RNA targeting OGT (shOGT) and non-targeting shRNA control vectors (Weinan Biotechnology Co., Ltd), as well as HA-FoxO1 overexpression plasmids, and transfect ATDC5 and SW1353 cells. The day before transfection, seed HEK293T cells at a density of 2 × 10^4^ cells per well in a 96-well plate. Use 100 µL of DMEM high-glucose medium supplemented with 10% FBS for culturing. Incubate the cells in a 5% CO_2_ incubator at 37 °C. On the day of transfection, prepare the transfection mix according to the following scheme:

Solution A: combine the Reporter plasmid (100 ng), Overexpression plasmid (100 ng), mOGT1 Expression plasmid (100 ng), and Internal reference plasmid (20 ng) in a total volume of 10 µL Opti-MEM. Solution B: dilute Lipofectamine 2000 in 10 µL Opti-MEM to a final volume of 0.32 µL. Allow the solutions to sit at room temperature for 5 min. Mix solutions A and B, then incubate at room temperature for 20 min. After adding 20 µL of the transfection complex to each well, continue to incubate the cells in a 5% CO_2_ incubator at 37 °C.

### Cell migration assay

Place Ibidi inserts in a 12-well plate as a migration barrier and seed ATDC5 cells into the inserts. Once the cells have adhered and fully grown, remove the inserts, leaving a gap between the two wells as a cell-free zone. Use an inverted microscope to record cell migration into the free zone at 0 and 24 h. Quantify the migration area using ImageJ, measuring the migration length to calculate the migration rate.

### Mass spectrometry (MS)

HA-FoxO1 protein complexes were extracted from 293 cells co-transfected with HA-FoxO1 and OGT using immunoprecipitation with HA magnetic beads. For liquid chromatography coupled with high-energy collisional dissociation tandem MS analysis, the sample underwent acetone precipitation, redissolution in urea, TCEP and IAA treatment for reduction and alkylation, and overnight trypsin digestion on a shaker. Following digestion, the peptides were desalted and subjected to gradient elution using C18 columns (Phenomenex, 15 μm, 300 A). For O-GlcNAc glycopeptide enrichment, peptides were redissolved and treated with an O-GlcNAc-specific antibody RL2. The enriched glycopeptides underwent gradient elution, concentrated to dryness under vacuum, and finally redissolved in ultrapure water. The entire three-phase column system (Analytical column, Captive column, Mobile phase) was integrated with a Dionex Ultimate 3000 RSLCnano system (ULTIM3000RSLCNANO, Thermo Fisher Scientific, MA, USA). Upon elution from the column, peptides traversed the ion transfer conduit, stabilized at 300 °C with an ionizing voltage of 1.9 kV, before their direct entry into the Orbitrap Exploris 480 mass spectrometry device (Thermo Fisher Scientific). The primary MS analysis was conducted with an m/z range of 700–2000 and a mass resolution of 60 k at m/z 200, ensuring adequate ion collection. This was followed by MS/MS with a mass resolution of 30 k, leading to data-dependent acquisition with the top 20 most intense ions. The HCD collision energy was normalized with stepped NCEs set at 30%. The data were processed against the human protein sequence database using Proteome Discoverer 2.4 software for database matching and protein identification.

### Alcian blue staining

Micromass cultivation was executed in accordance with prior documentation [40]. Briefly, ATDC5 cells were resuspended in DMEM/F12 at a density of 3 × 10^7^ cells/ml. Fifty microliters of the cell suspension was placed in each well of a 12-well plate and incubated at 37 °C for 2 h, after which chondrogenic differentiation medium was introduced and maintained at 37 °C for a 14-day period. After differentiation, ATDC5 cells were extensively rinsed with PBS to eliminate any loosely attached cells or debris, followed by fixation using a 4% paraformaldehyde solution. Once stabilized, an extended staining process was performed using 0.3% Alcian blue (GP1040, Servicebio, Wuhan, China) through the night. Conduct quantitative analysis using ImageJ software.

### Flow cytometric analysis

Following a 5-min centrifugation at 1000 RPM. Suspend ATDC5 and SW1353 cells in binding buffer to achieve a concentration of 1 × 10^6^ cells/ml. Stain the cells using the FITC-labeled Annexin V Apoptosis Detection Kit (555335, BD, NJ, USA) according to the manufacturer’s protocol. Analyze the stained cells using a flow cytometer. Based on the staining results of Annexin V and PI, distinguish live cells, early apoptotic cells, and late apoptotic/necrotic cells, and quantify each cell population.

### TUNEL staining

Frozen sections of facet joint cartilage were rinsed twice with PBS. Subsequently, the sections underwent permeation with 20 μg/ml of proteinase K for a duration of 10 min. Following a 5-min PBS wash, the sections were subjected to a 4% formaldehyde solution for 5 min. After a rinse and a brief acclimation at ambient temperature spanning 5–10 min, the specimens received the TdT reaction concoction. This mixture settled at 37 °C inside a humidity-regulated obsidian compartment for 60 min. To terminate the reaction, the sections were dipped in 2 × SSC for 15 min. A final wash was given before counterstaining with DAPI for nuclear staining. The local red fluorescence of the apoptotic tissues was subsequently brought into focus with a laser-assisted confocal microscopy device (PCM 200, Nikon).

### Micro-CT

Prior to histological evaluation, L5 facet joints underwent analysis using precision-enhanced micro-CT (Skyscan1275). Scan configurations were established at a voltage of 75 kV, a current of 46 μA, and a pixel clarity of 12 μm. For the reassembly of images and intricate morphological evaluation, the optimal tools were NRecon v1.6 and CTAn v1.15 software. The tri-dimensional visuals were rendered using the CTVol v2.2 visualization tool. We selected coronal views of the L5 SAP subchondral bone for three-dimensional histomorphometric evaluations. Parameters, including bone quantity, the proportion of bone volume to trabecular volume (BV/TV), the ratio of bone surface to bone volume (BS/BV), average Tb.Th. and typical trabecular spacing (Tb.Sp.) within the L5 SAP subchondral skeletal region were evaluated.

### Catwalk analysis

To gauge the influence of OGT removal in L4/5 and L5/6 facet joint cartilage on rodent mobility, a CatWalk XT 10.0 stride assessment mechanism was employed to record the gait information of the animals. This evaluation was conducted to the mice aged at 8 months post-injection of AAV2-NC or AAV2-cre. A dozen mice (*n* = 6 for each set) underwent stride evaluation. Each rodent was situated on one end section of the glass walkway of the system, contrasted by a dark synthetic backdrop. Beneath the walkway, rapid-capture video device was mounted to document the full traversal journey of the mice. Key parameters recorded for subsequent analyses included mean intensity, swing speed, stride length, and stand time. The mean intensity is the average intensity of the paw prints, providing an indication of the pressure exerted by the paw during contact with the glass surface. The swing speed represents the speed at which an animal paw moves through the air from the backswing to the forward swing. It is a measure of the speed of the non-contact phase of the gait cycle. The stride length refers to the distance covered from the point of initial contact of one paw to the point of subsequent contact of the same paw. It is a measure of the distance covered in one complete cycle of a leg movement during locomotion. Stand time, also known as stance time, refers to the duration of time that a paw is in contact with the ground during a gait cycle. It is the period from the initial contact of the paw with the ground to the point where the paw lifts off the ground again. It provides information about the weight-bearing phase of the gait cycle.

### RNA-seq and bioinformatic analysis

Collect cartilage from the spinal facet joints of FoxO1^L/L^ and FoxO1^L/L^ Col2a1-creERT mice, as well as SW1353 samples from the shOGT, CON, and OGT groups for RNA sequencing (RNA-seq). Total RNA was treated with the Ribo-Zero Gold rRNA Removal Kit (Illumina) to eliminate ribosomal RNA. Libraries were sequenced on an Illumina NovaSeq 6000 platform to generate ≥30 million paired-end 150-bp reads per sample, ensuring robust transcriptome coverage. For RNA-seq differential expression analysis, we applied the Benjamini-Hochberg procedure to control the false discovery rate. DEGs were defined using DESeq2 with thresholds of |log2(fold change)| > 1 and adjusted *p* < 0.05. Genes with a *q*-value threshold set at 0.05 and a fold-change exceeding 2 or falling below −2 are defined as DEGs.

Genes exhibiting differential expression and sharing commonalities were depicted using the Venny 2.1.0 online platform in the form of a Venn diagram. The sequencing information has been archived in the SRA database under the accession number SUB13701195 (PRJNA997582).

### Proliferation assay

In 96-well plates, ATDC5 cells were dispensed at a density of 2 × 10^5^ cells/ml. Following a 24-h incubation with 50 μM EdU, cell growth was evaluated employing the EdU proliferation Kit. Visualization of ATDC5 cells was carried out using a Nikon ECLIPSE Ni-E fluorescence microscope running on the NIS-Elements D 5.11 platform. The quantification of cell populations was performed with ImageJ Fiji software, and the proliferation rate was established through comparing the count of EdU-labeled ATDC5 to the all.

### Luciferase assay

On the day prior to transfection, HEK293T cells were plated at a density of 2 × 10^4^ cells per well in a 96-well plate, with 100 µL of high-glucose DMEM supplemented with 10% FBS in each well, and incubated in a 37 °C, 5% CO_2_ incubator. On the transfection day, the transfection mix was prepared with Solution A containing 100 ng each of the reporter, overexpression, and mOGT1 expression plasmids, plus 20 ng of the housekeeping gene plasmid in 10 µL of Opti-MEM. Solution B contained 0.32 µL of Lipofectamine 2000 in 10 µL of Opti-MEM. Both solutions were left to stand at room temperature for 5 min before being mixed and incubated for an additional 20 min at room temperature. Then, 20 µL of the mixed transfection solution was added to each well, and the cells were incubated in a 37 °C, 5% CO_2_ incubator.

### Statistical analysis

Results are presented as mean ± SEM. Comparisons between two groups were made using the two-tailed independent t-test, and multiple group analyses were conducted with one-way ANOVA.

For experiments involving multiple groups, we performed Tukey’s post hoc test following one-way ANOVA to adjust for multiple comparisons. Adjusted *p*-values are now explicitly reported in figure legends and results. All figures and tables now distinguish between raw *p*-values (for single comparisons) and adjusted *p*-values (for multiple comparisons). Statistical relevance was denoted by a *p*-value below 0.05.

## Supplementary information

Supplementary File

## Data Availability

The datasets used and/or analyzed during the current study are available from the corresponding author on reasonable request.
