# Supplementary material for: O-GlcNAc transferase influences the progression of degenerative cartilage disease in lumbar facet joint osteoarthritis through FoxO1 and EGR1
Source: Cell Death Discov. 2025 Oct 16;11:462. doi: 10.1038/s41420-025-02732-1 (PMC12533218; doi:10.1038/s41420-025-02732-1)
Supplement: Supplementary file 1 — Supplementary File [file 41420_2025_2732_MOESM1_ESM.docx]

**Figure S1.**

Immunofluorescence staining of facet joint sections of AAV2-NC and AAV2-cre group. Blue indicates DAPI, green indicates OGT, and red indicates RL2. Scale bars indicate 100 μm. Uncertainty markers: SEM. N = 3. *, P < 0.05. **, P < 0.01.

**Figure S2.**

Through LC-HCD-MS/MS, we detected the spectra and deciphered the sequencing of the peptide (O-GlcNAc-bearing 620-646) at [M+H] m/z 1094.83948 Da. This method's unique capabilities enabled the identification of both b- and y- ions via mass analysis. The observed spectral peaks matched the m/z values of specific fragment ions. Comparative tables delineate the predicted m/z values for these ions, accentuating the results from the LC-HCD-MS/MS in a crimson shade.

**Figure S3.**

OGT regulates the ubiquitination level of FoxO1 through O-GlcNAc glycosylation modification, the full, uncropped Western blots are available as Supplementary Figure S11.

**Figure S4.**

HA-tagged versions of wild-type FoxO1 (WT) and its Alanine substitution mutants were expressed in HEK 293T cells with or without exogenous OGT expression. Cell lysates were analyzed via immunoblot for O-GlcNAcylation (using anti-O-GlcNAc RL2) and total FoxO1 (using anti-HA). Notably, the FoxO1 4A mutant (with substitutions at T317, S550, T648 and S654) lacked O-GlcNAcylation. The T627 mutant, when compared to the WT, didn't exhibit significant differences in O-GlcNAcylation, but when combined with the FoxO1 4A, its O-GlcNAcylation was noticeably reduced compared to the FoxO1 4A alone. This result suggests that the FoxO1 residue T627, when mutated along with 4A, can have a synergistic effect, significantly reducing the O-GlcNAcylation of FoxO1. The full, uncropped Western blots are available as Supplementary Figure S11.

**Figure S5.** **Analysis via QRT-PCR evaluated the expression of target genes influenced by OGT/FoxO1 and several posttranslational alterations in FoxO1**

Quantitative PCR analysis of ACAN, Col2a1, and IL-6 expression in ATDC5 cells transfected FoxO1, FoxO1- 4A, or FoxO1-4A+T627A for 48 h. GAPDH is used as an internal control. Uncertainty markers: SEM. *, P < 0.05. **, P < 0.01.

**Figure S6.**

(A) The relative mRNA levels of EGR1 qualified by qRT-PCR in CON, shOGT, and FoxO1 KO group. (B) Gene abundances of EGR1 in ATDC5 cells transfected with control, shOGT, and shOGT plus FoxO1-overexpressing lentivirus. (C) Quantitative PCR analysis of EGR1 expression in ATDC5 cells transfected FoxO1, FoxO1-4A or FoxO1-4A+T627A for 48 h. GAPDH is used as an internal control. Uncertainty markers: SEM. *, P < 0.05. **, P < 0.01. ***, P < 0.001. For the specified comparison, no significant difference was observed (N.S.).

**Figure S7.**

Complete and uncropped western blots in Figure 1

**Figure S8.**

Complete and uncropped western blots in Figure 3

**Figure S9.**

Complete and uncropped western blots in Figure 4.

**Figure S10.**

Complete and uncropped western blots in Figure 7.

**Figure S11.**

Complete and uncropped western blots in Figure S3, S4.

**
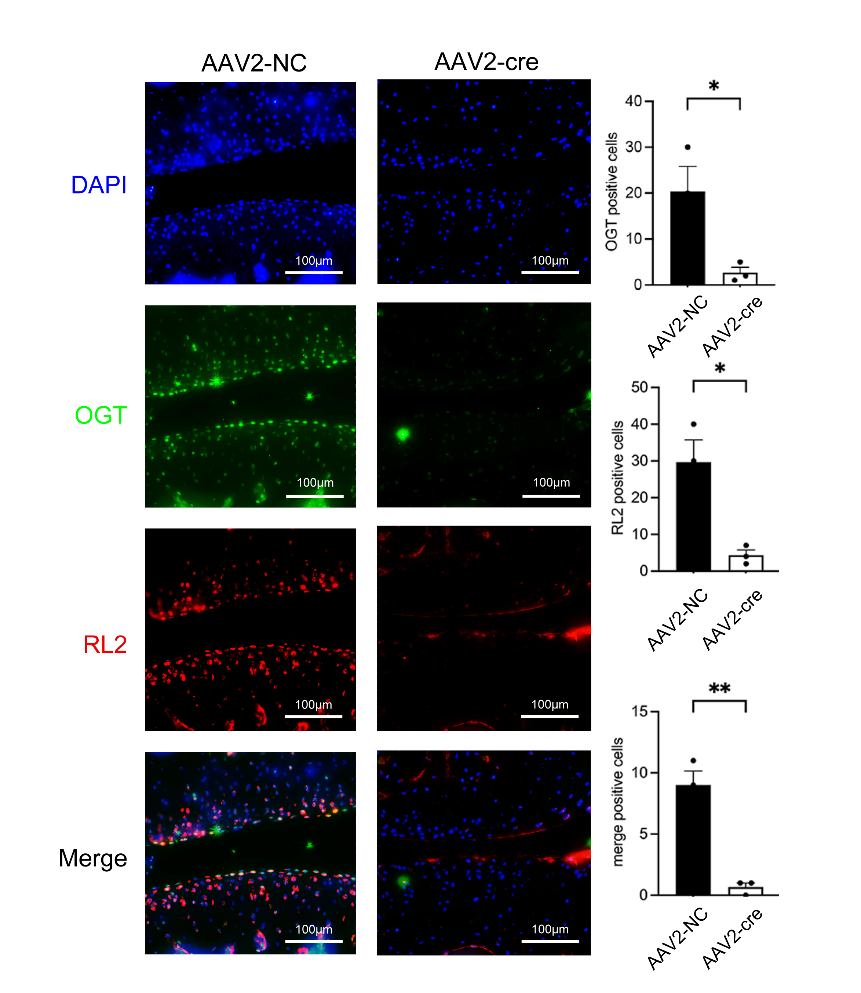
**

**Figure S1.**

**
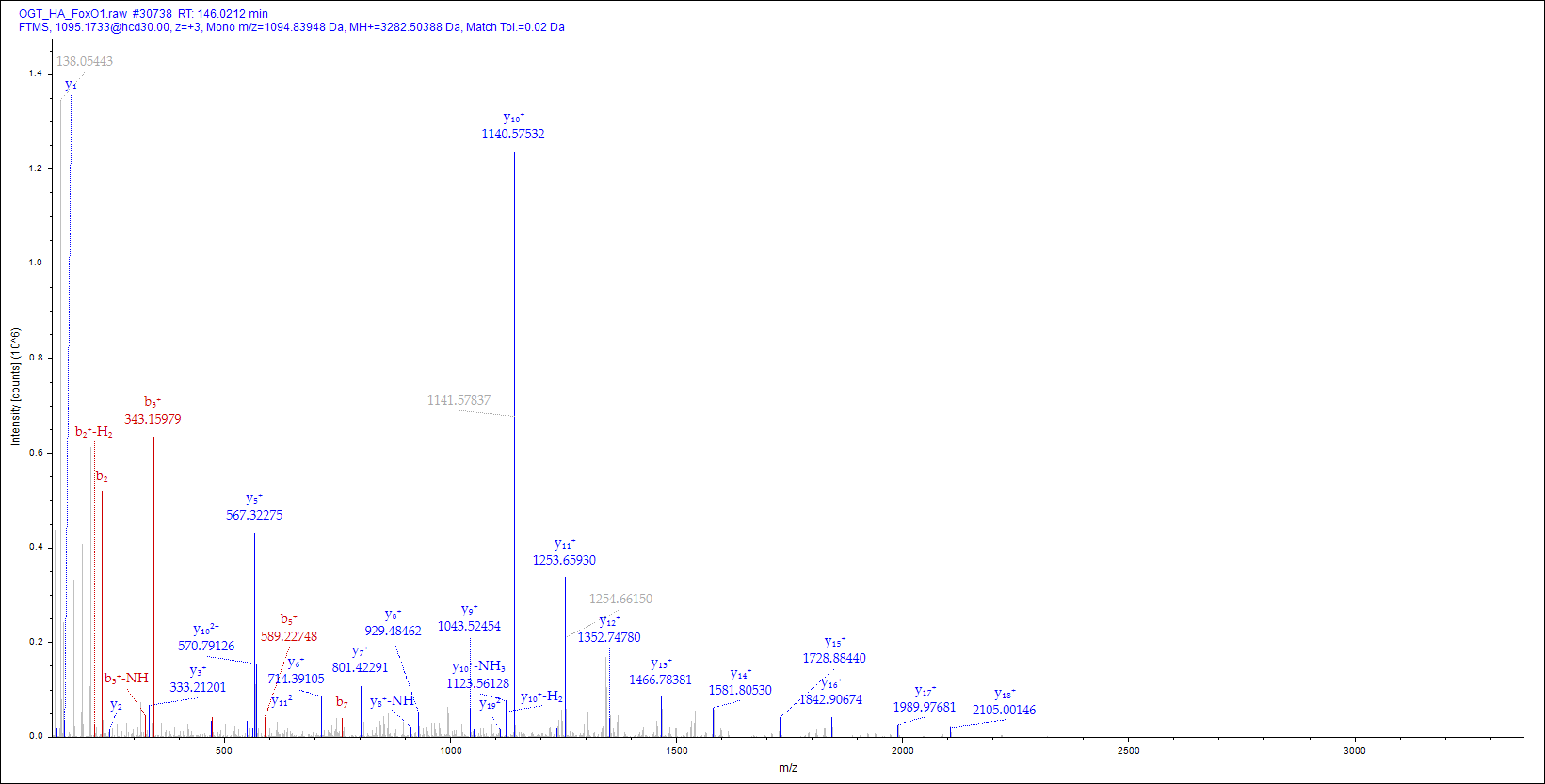
**

**Figure S2.**

**
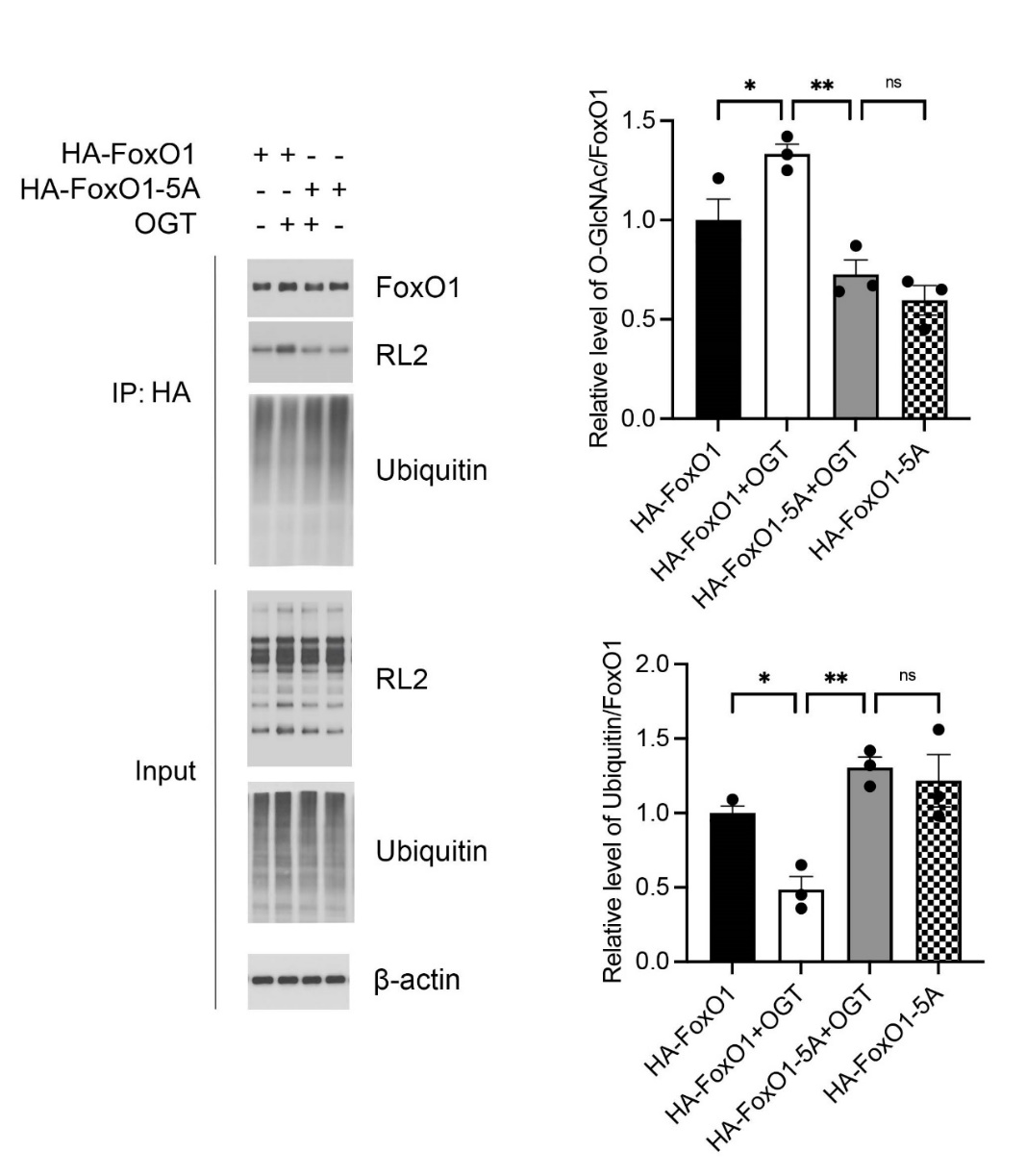
**

**Figure S3.**

**
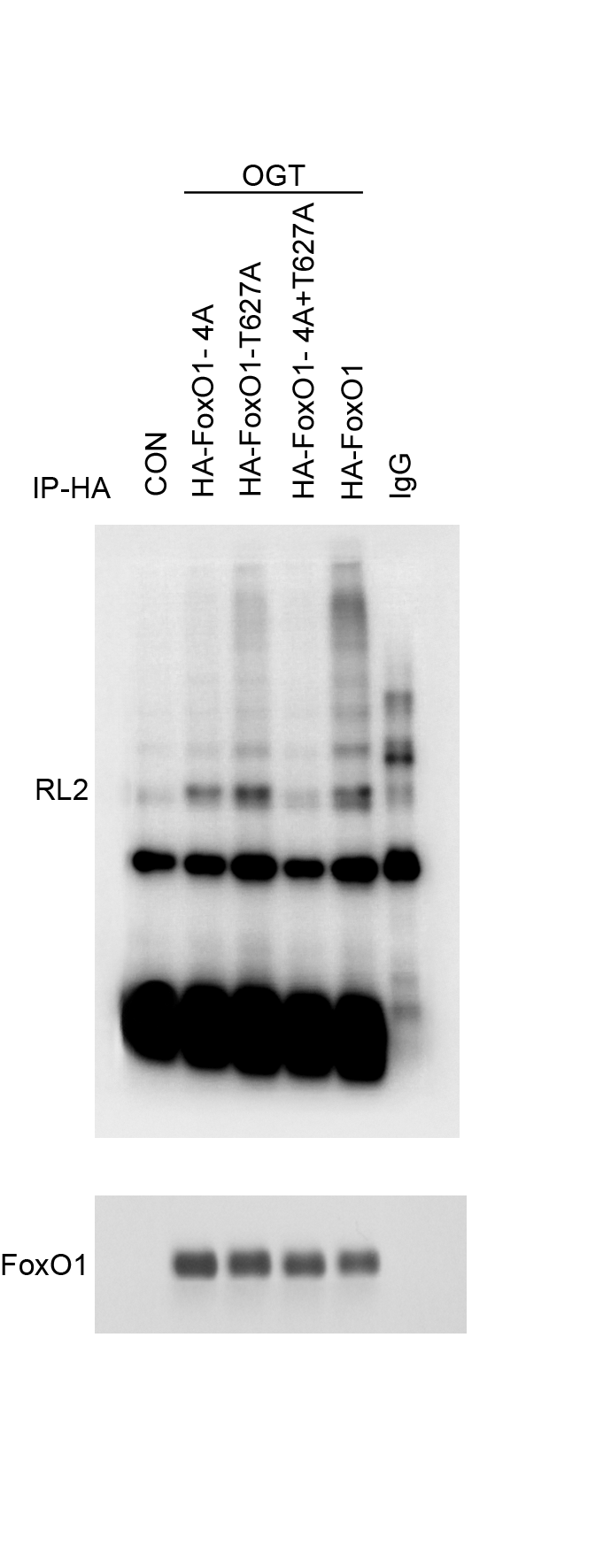
**

**Figure S4.**

**
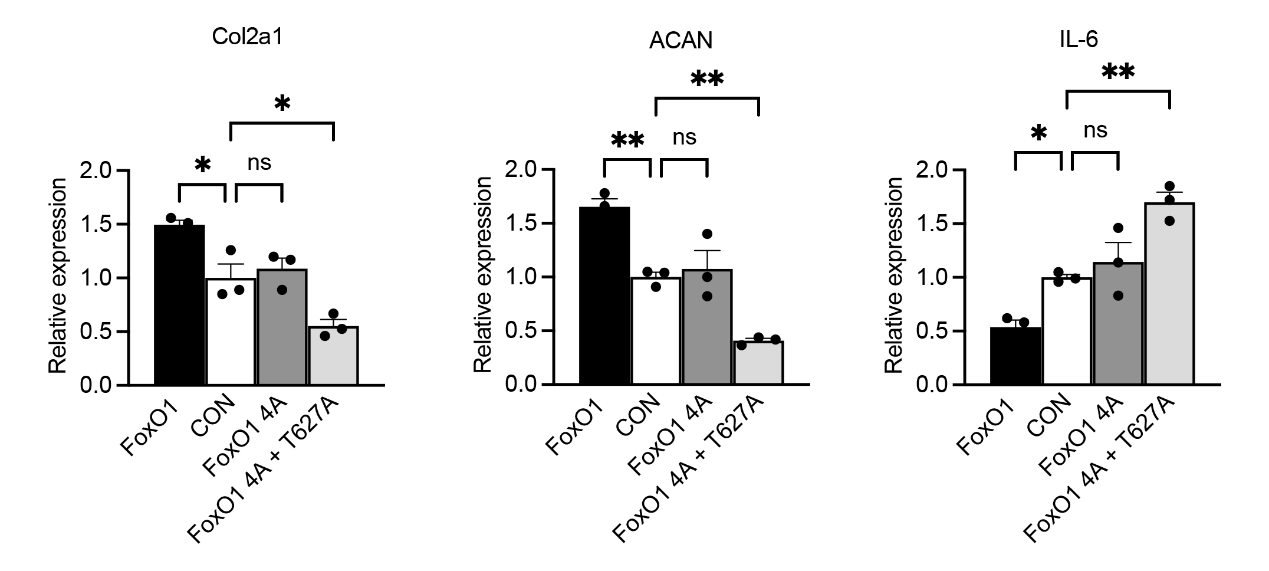
**

**Figure S5.**

**
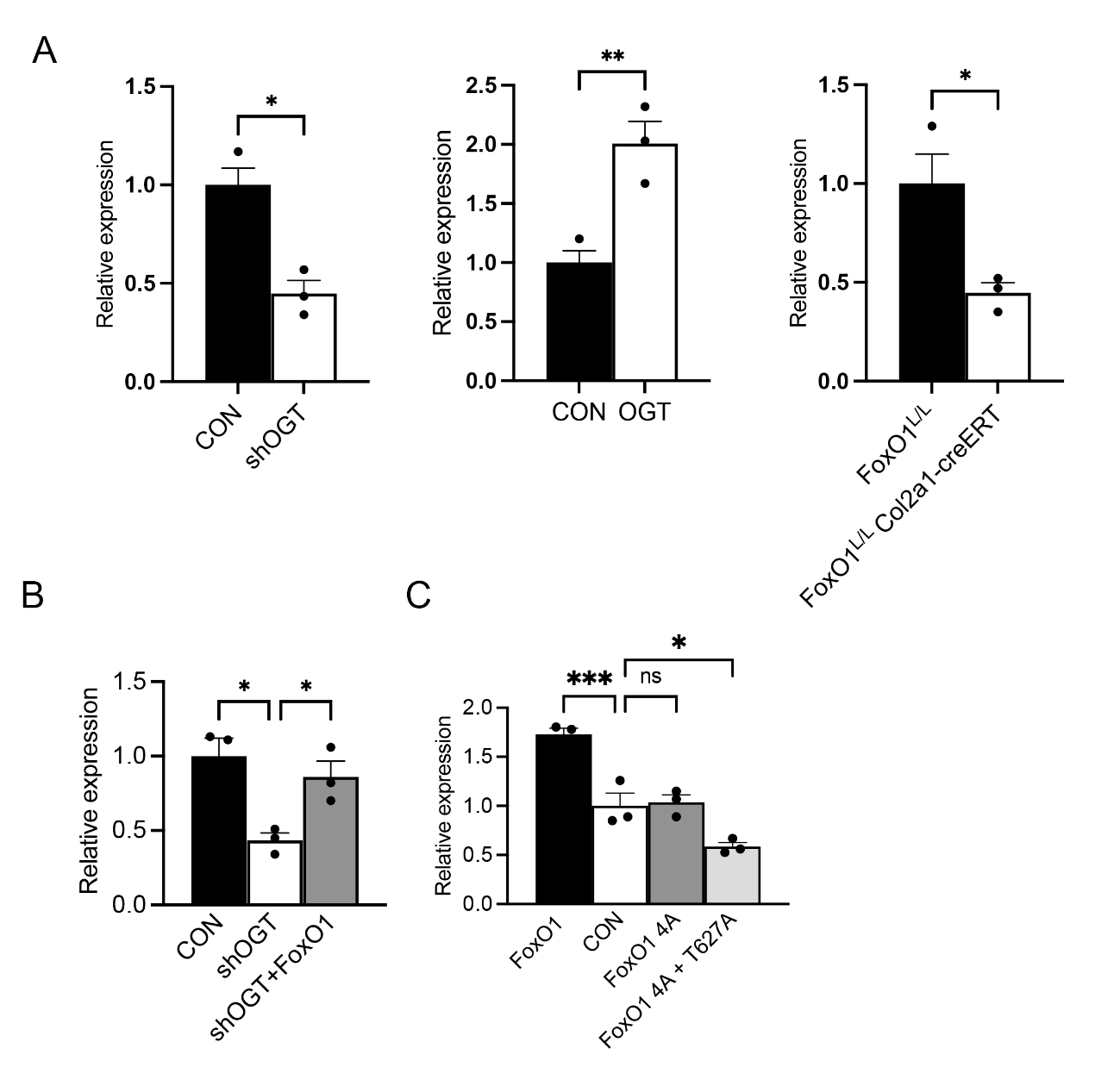
**

**Figure S6.**

**
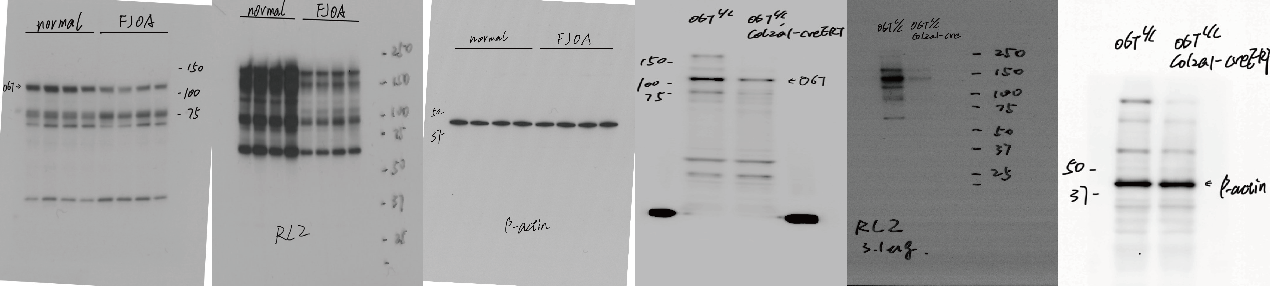
**

**Figure S7.**

**
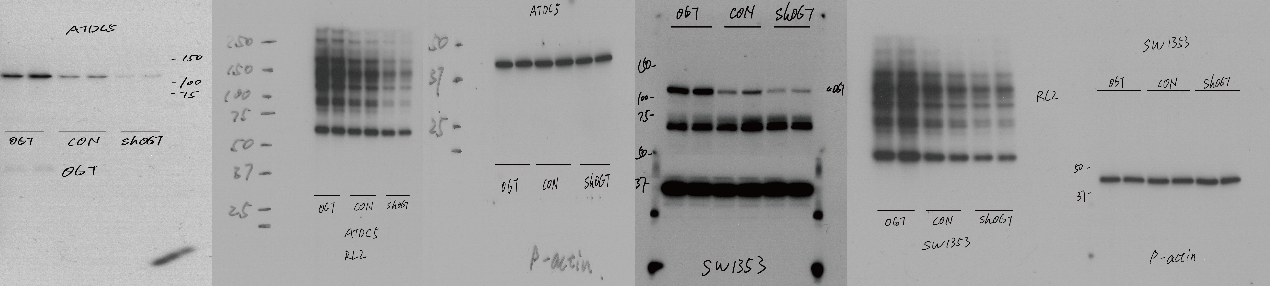
**

**Figure S8.**

**
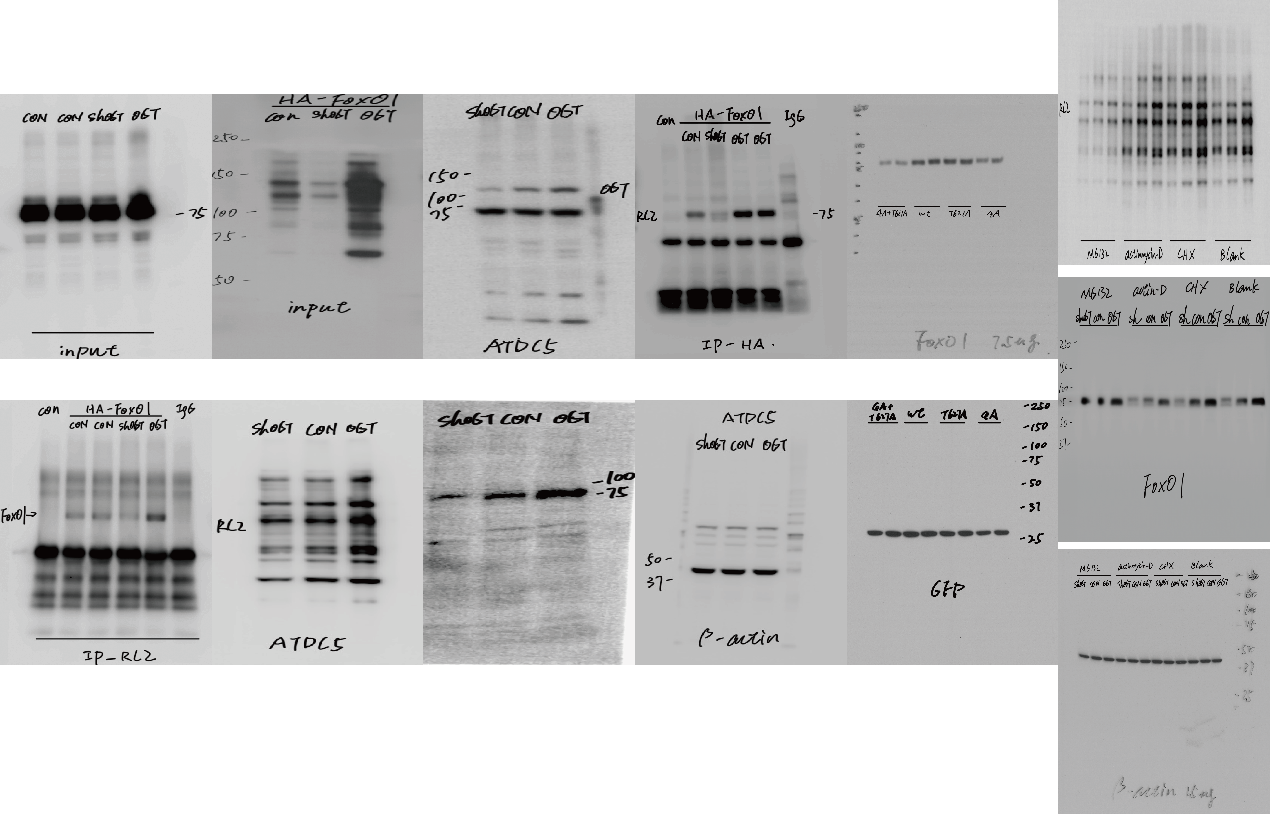
**

**Figure S9.**

**
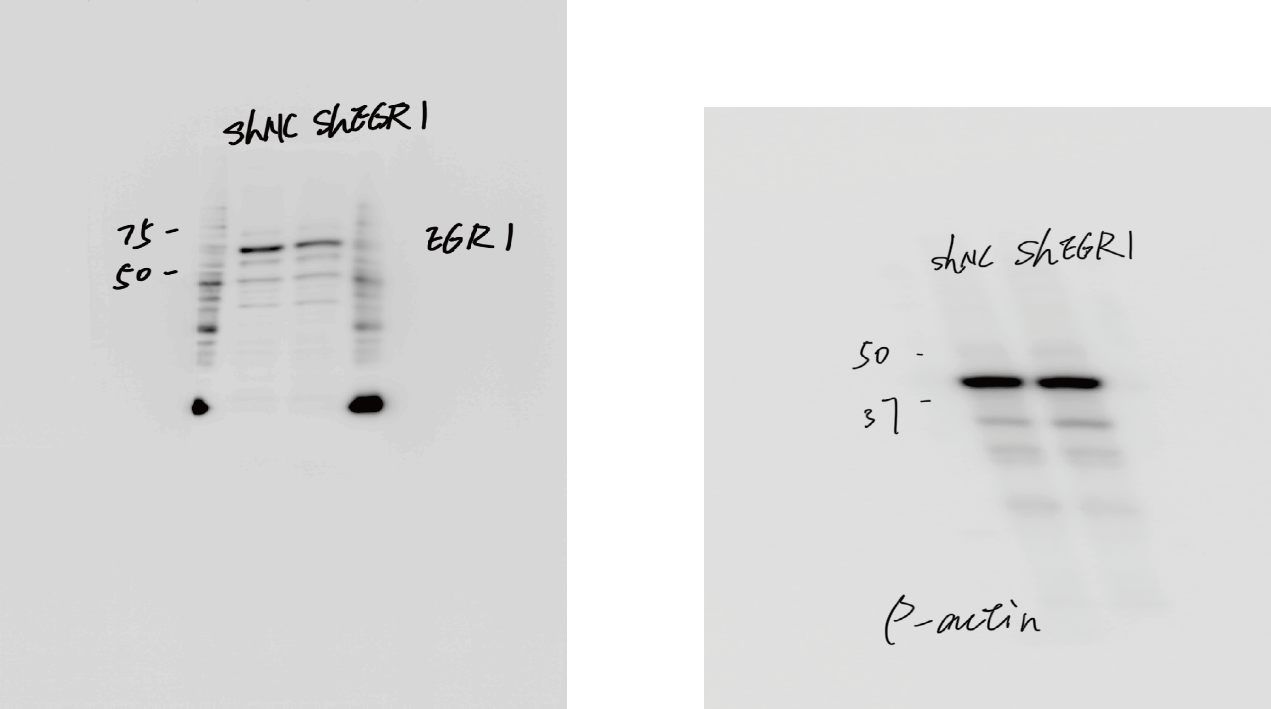
**

**Figure S10.**

**
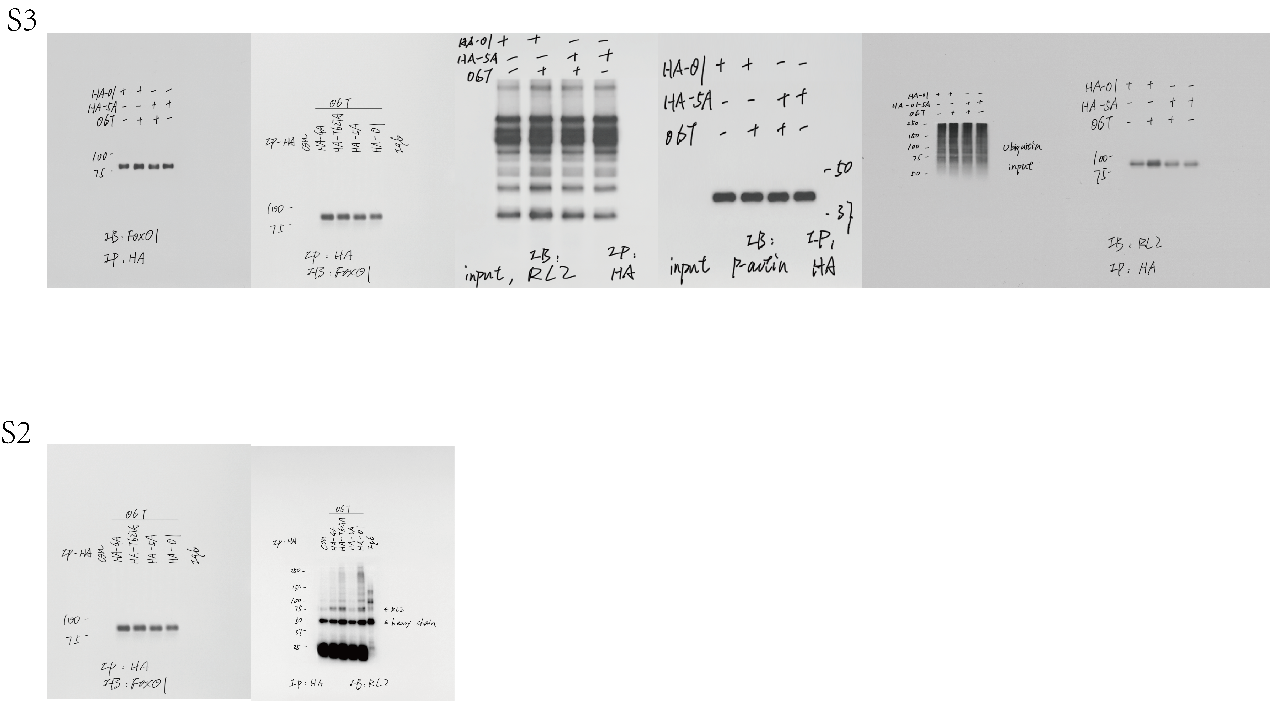
**

**Figure S11.**
